# Towards Continuous Camera-Based Respiration Monitoring in Infants

**DOI:** 10.3390/s21072268

**Published:** 2021-03-24

**Authors:** Ilde Lorato, Sander Stuijk, Mohammed Meftah, Deedee Kommers, Peter Andriessen, Carola van Pul, Gerard de Haan

**Affiliations:** 1 Department of Electrical Engineering, Eindhoven University of Technology, 5612 AZ Eindhoven, The Netherlands; s.stuijk@tue.nl (S.S.); G.d.Haan@tue.nl (G.d.H.); 2 Department of Family Care Solutions, Philips Research, 5656 AE Eindhoven, The Netherlands; mohammed.meftah@philips.com; 3 Department of Neonatology, Maxima Medical Centre, 5504 DB Veldhoven, The Netherlands; Deedee.Kommers@mmc.nl (D.K.); P.Andriessen@mmc.nl (P.A.); 4 Department of Applied Physics, Eindhoven University of Technology, 5612 AZ Eindhoven, The Netherlands; C.vanPul@mmc.nl; 5 Department of Clinical Physics, Maxima Medical Centre, 5504 DB Veldhoven, The Netherlands

**Keywords:** thermal camera, respiration, infants, unobtrusive, vital signs, camera, thermography, infrared, NICU, non-nutritive sucking

## Abstract

Aiming at continuous unobtrusive respiration monitoring, motion robustness is paramount. However, some types of motion can completely hide the respiration information and the detection of these events is required to avoid incorrect rate estimations. Therefore, this work proposes a motion detector optimized to specifically detect severe motion of infants combined with a respiration rate detection strategy based on automatic pixels selection, which proved to be robust to motion of the infants involving head and limbs. A dataset including both thermal and RGB (Red Green Blue) videos was used amounting to a total of 43 h acquired on 17 infants. The method was successfully applied to both RGB and thermal videos and compared to the chest impedance signal. The Mean Absolute Error (MAE) in segments where some motion is present was 1.16 and 1.97 breaths/min higher than the MAE in the ideal moments where the infants were still for testing and validation set, respectively. Overall, the average MAE on the testing and validation set are 3.31 breaths/min and 5.36 breaths/min, using 64.00% and 69.65% of the included video segments (segments containing events such as interventions were excluded based on a manual annotation), respectively. Moreover, we highlight challenges that need to be overcome for continuous camera-based respiration monitoring. The method can be applied to different camera modalities, does not require skin visibility, and is robust to some motion of the infants.

## 1. Introduction

Vital signs need to be monitored in specific hospital environments. Infants, in particular, may need continuous monitoring when admitted to neonatal wards like Neonatal Intensive Care Units (NICUs). Commonly monitored vital signs include heart rate, Respiration Rate (RR), blood oxygen saturation, and skin temperature. Respiratory instability in infants is one of the main reasons for admission. Therefore, respiration is monitored in neonatal wards to detect critical situations, i.e., apneas (sudden cessations of breathing). If leading to hypoxia, these events can result in long-term or permanent impairment [1], and therefore, the detection of apneas is crucial.

The monitoring of respiration, but in general of most vital signals, requires attaching electrodes and sensors to the infants’ skin, which can be uncomfortable for the infants or even cause skin damage [2]. Moreover, impedance pneumography or Chest Impedance (CI), which is commonly used in neonatal wards for respiration monitoring, is not very reliable in apnea detection [3].

For these reasons, unobtrusive solutions are being investigated for both hospital environments and home-care. Respiration motion can be detected using RGB (Red Green Blue) or Near-Infrared (NIR) cameras [4,5,6], radars [7,8,9], or pressure-sensitive mats [10,11,12]. Solutions using thermal cameras as in Mid-Wave Infrared (MWIR) or Long-Wave Infrared (LWIR) have also been investigated [13,14,15]. Thermal cameras can detect both respiration motion and respiratory flow, which can be useful in the detection and identification of apnea episodes in infants since obstructive apneas and mixed apneas still present respiratory effort, i.e., motion, but no flow [16].

Motion artifacts are a major problem for both the current monitoring technologies, e.g., CI, and most of the non-contact solutions [17,18]. Motion robustness is, therefore, paramount when aiming at a continuous RR detection in infants. Moreover, since lethargy (hypotonia and diminished motion) and seizures (epileptic insult, repetitive motion activity) are associated with serious illnesses of the newborn [19,20], motion is an important vital sign, that has also been linked to the prediction of apnea and neonatal sepsis [21,22].

Multiple works proposed solutions to tackle the motion artifacts or random body movement problem in camera-based respiration detection [23,24]. However, not all random body movements hide the respiration information and by excluding all the segments containing motion from the respiration monitoring step, potentially usable segments are also excluded. In a recent study published by Villarroel et al. [25] motion robustness was achieved by combining an indicator of the quality of the reference signal with an indicator of the agreement between the RRs obtained using different sources. However, the detection of the respiration signals is dependent on skin visibility. Infants who are cared for in open beds in neonatal wards or in home-care environments are usually covered with blankets and wear clothing. A solution based on skin visibility, particularly of the chest/torso area, would, therefore, be impractical for these cases.

Therefore, extending our previous work [26], which estimated the RR in static moments extracted from infants’ thermal videos, in this paper, we analyze the performance of our algorithm in challenging conditions containing various types of motion, also semi-periodic ones such as Non-Nutritive Sucking (NNS). We aim at achieving motion robustness by ensuring that the RR can be accurately estimated also in the presence of some motion, e.g., head and limbs movements. We achieve this using a motion detector optimized to detect specifically the kind of motion hiding the respiratory information, which often cause impaired CI reference signal as well. This algorithm was trained and tested on thermal and RGB videos, both video types were acquired on different infants, i.e., the babies in the thermal videos are different from the babies in the RGB videos. In total, the thermal dataset includes around 42 h of videos recorded on fifteen infants in a neonatal ward. The RGB dataset is smaller and includes 50 min of video recorded on two infants. We, therefore, prove that both our motion detector and our RR estimation algorithm with improved motion robustness can be used for both visible and thermal modalities, without the need of skin visibility. To our knowledge, this is the first work showing results on such a large dataset of neonatal thermal recordings for respiration monitoring.

The remaining of this paper is organized as follows: Section 2 describes the method developed and explains the setup used and the dataset. Section 3 and Section 4 present the results obtained and the discussion, respectively. Section 5 contains the conclusions of this work.

## 2. Materials and Methods

### 2.1. Materials

#### 2.1.1. Experimental Setup

Two different setups were used to collect the RGB videos and thermal videos used in this work. The thermal videos were collected using three thermal cameras positioned around the infants’ bed. The cameras used are FLIR Lepton 2.5, they are sensitive in the LWIR range, the resolution is 60×80 pixels, the thermal sensitivity is 50 mK, and the average frame rate is 8.7 Hz. The acquisition was performed using MATLAB (MATLAB 2018b, The MathWorks Inc., Natick, MA, USA). Due to the acquisition strategy, the 3 h of recording are split into 9 videos of 20 min each, gaps of up to 4 s can be present between the videos. For further information on the setup refer to [26].

The visible images were obtained in a separate data collection with a single RGB camera (UI-2220SE, IDS), that was positioned on a tripod to visualize the baby in the open bed. Some videos were collected from the side and others from the top. The frame rate and resolution are, respectively, 20 Hz and 576×768 pixels. In both cases, the reference CI signal sampled at 62.5 Hz was collected using the patient monitor (Philips MX800). To solve the synchronization problem, an artifact (simultaneously disconnecting the CI leads and covering the view of one of the cameras) was generated at the start of each recording to synchronize CI and videos.

#### 2.1.2. Dataset

The dataset was split into two sets, one called the training and testing set, which is used to optimize and test the motion detection step, and adjust our respiration monitoring algorithm. The other one called the validation set will be used to obtain unbiased results for both the motion detection step and the RR detection step. Table 1 contains the infants’ data and the duration of the recordings for the training and testing set, and the validation set. The infants were assigned to the two sets based on the availability of the data. The thermal videos amount to a total of around 42 h acquired on fifteen infants, all the infants were monitored for around 3 h except for infant 7, which has a total video duration of around 1 h, due to setup problems.

The RGB videos of infant 8 and 9 amount to a total video duration of around 52 min.

Both thermal and RGB datasets were collected in the Medium Care Unit of the neonatal ward in the Maxima Medical Centre (MMC) in Veldhoven, The Netherlands. Both studies received a waiver from the ethical committee of MMC (the thermal dataset with ID N19.074 and the RGB dataset with ID N12.072), and informed consent was obtained from the infants’ parents prior to the study.

#### 2.1.3. Manual Annotation

One of the authors annotated the videos contents, including motion occurrences, and it was then used as ground truth for the motion detection step. A MATLAB built-in application called *Video Labeler* was used to annotate the videos. A set of labels was defined to describe the possible visible events, the labels are not exclusive, meaning that multiple labels can be true at the same time. We defined two classes of motion type 1 and type 2 motion. The labels are presented in Table 2.

The main difference between the two types of motion, i.e., 1 and 2, is the involvement of the chest in the motion event. Type 1 is a motion that involves the chest/torso area, where the respiration motion can be usually seen. In our classification this is, therefore, considered as the kind of motion that results in hiding the respiration information, which can cause artifacts also in the CI reference signal. Type 2, instead, does not involve chest or torso movements but affects other parts as, head, hands, arms, fingers, or even facial expressions.

**Figure 1:**
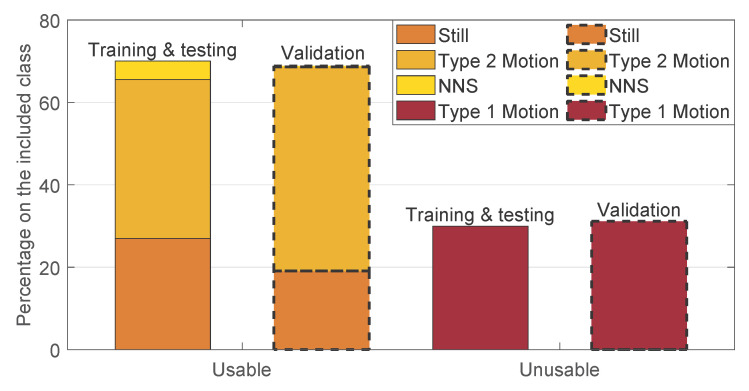
Results of the manual annotation: the breakdown of the included class into the subcategories for the training and testing, and the validation set.

The segments of videos including events labeled as categories iii and iv in Table 2 were excluded in this work, since they would require different detectors, e.g., interventions detection or infant presence detection [25]. In particular, the included and excluded percentages in the entire dataset are, respectively, 73.86% and 26.14%. The majority of the excluded moments are caused by the babies being out of bed and by interventions, 46.4% and 31.8%, respectively. The breakdown of the included moments are shown in Figure 1 split between the training and testing, and the validation set. The segments containing type 1 motion events are considered unusable for the estimation of the RR, whereas, the ones containing type 2 motion, still, and NNS are considered usable. The cumulative percentages of type 2 motion, still, and NNS constitute 70.03% and 68.85% of the included moments for the training and testing, and the validation set, respectively. The remaining part contains the fragments annotated with type 1 motion. The occurrence of type 1 motion is, therefore, very similar between the two sets.

### 2.2. Method

**Figure 2:**
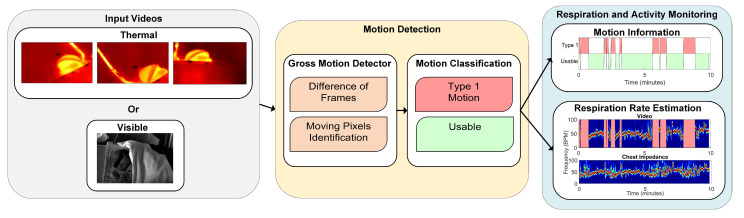
Main blocks of the processing chain and an example of the results.

The algorithm proposed in this work can be split in two main parts, i.e., motion detection and RR estimation. The first was designed to detect type 1 motion, since segments containing type 2 motion are considered usable for the RR detection and it is, therefore, not necessary to detect their occurrence. Therefore, if type 1 motion was detected the RR could not be accurately estimated and an indication that the baby was moving was provided. Otherwise, the video segment did not contain type 1 motion and it was classified as usable and the RR was estimated using the second part of our algorithm. These steps are shown in Figure 2. The algorithm was implemented using MATLAB.

#### 2.2.1. Preprocessing

The thermal videos were linearly interpolated to compensate for the acquisition strategy, which resulted in a non-uniform sampling rate, because external triggering was not used. A 1D interpolation was applied to each pixel’s time domain signal, using the MATLAB function *interp1*, the result was three videos sampled at 9 Hz, close to the average frame rate, with a resolution of 60×80 pixels. The RGB data were converted to grayscale (using the MATLAB function *rgb2gray*) and downscaled, to allow faster processing, with a downscale factor of 3 resulting in a final video resolution of 192×256. The grayscale videos were also temporally downsampled to reach the same sampling rate as the thermal videos, i.e., 9 Hz, from an initial sampling frequency of 20 Hz, for faster processing. The frame sizes will be indicated as $\tilde{M}\times \tilde{L}$, which will correspond to 60×80 in the thermal case and 192×256 in the visible case.

A sliding window approach was used for both the motion detection and the RR estimation steps. Considering a trade-off between latency and frequency resolution and the fact that longer windows means more sliding windows may contain motion events, a relatively short window size of 8 s was chosen with a slide of 1 second.

#### 2.2.2. Motion Detection

- Gross Motion Detector: let $X(nT_{s})$ be the frames in the *j*th window, with $n=0+\left(j-1\right)/T_{s},\;1+\left(j-1\right)/T_{s},\;\ldots ,\;N+\left(j-1\right)/T_{s}$, and N=72 samples, corresponding to the samples in the *j*th window with a sampling period $T_{s}=0.111$ s. The gross motion detector was based on the absolute value of the Difference of Frames (DOFs) in the *j*th window. More formally:
  (1) $$D\left(uT_{s}\right)=\left|\frac{\partial X(nT_{s})}{\partial n}\right|,$$
  the $\frac{\partial }{\partial n}$ operator represents the partial derivative with respect to the time dimension. $D(uT_{s})$ contains the frames resulting from the absolute value of the difference of frames operation at each time sample, with $u=0+\left(j-1\right)/T_{s},\;1+\left(j-1\right)/T_{s},\;\ldots ,\;\left(N-1\right)+\left(j-1\right)/T_{s}$. At this point, a first threshold value was introduced, which turns **D** into binary images identifying what we considered to be moving pixels:
  (2) $$\mathrm{MP}\left(uT_{s}\right)=\left\{\begin{array}{cc} 1 & \mathrm{if}\;D\left(uT_{s}\right)>thr_{1}\\ 0 & \mathrm{otherwise}. \end{array}\right.$$
  $thr_{1}$ is a threshold that was introduced to differentiate the source of the change between noise and motion, it is defined as:
  (3) $$thr_{1}=\frac{Range(X)}{f_{1}},$$
  the numerator represents the range of X, i.e., the difference between the maximum value and the minimum value considering all the pixels of all the frames in X, and $f_{1}$ is a value that was optimized. The ratio of moving pixels was then calculated as:
  (4) $$s\left(uT_{s}\right)=\frac{\sum_{\tilde{m}=1}^{\tilde{M}}\sum_{\tilde{l}=1}^{\tilde{L}}mp_{\tilde{m},\tilde{l}}\left(uT_{s}\right)}{\tilde{M}\cdot \tilde{L}}.$$
  Here, $mp_{\tilde{m},\tilde{l}}\left(uT_{s}\right)$ is an element of $\mathrm{MP}(uT_{s})$ at the position $\tilde{m}$ and $\tilde{l}$.
- Motion Classification: the ratio of moving pixels $s(uT_{s})$ was used to perform the classification between usable and unusable segments for RR detection. In particular, we aim at detecting the unusable moments, i.e., the ones containing type 1 motion. The main assumption is that type 1 is part of a more complex kind of motion, typical of infants’ crying motion. Therefore, the simplest way to detect it is to assume that type 1 motion will result in more moving pixels compared to any of the usable segments.
  To perform a classification between the two, a second threshold $thr_{2}$ was introduced, which was applied to the ratio of moving pixels $s(uT_{s})$. The final classification was, therefore, performed on a window-based fashion, i.e., each window was classified as containing type 1 motion, corresponding to 1, or usable, corresponding to 0.
  Since we used three cameras in the thermal setup, we applied this algorithm three times. For the RGB dataset this was not necessary, as there was only a single camera used. In the visible case the classification will be:
  (5) $$Motion_{vis}\left(j\right)=\left\{\begin{array}{cc} 1 & \mathrm{if}\;\exists \;\;u\;:\;s\left(uT_{s}\right)\geq thr_{2}\\ 0 & \mathrm{otherwise}. \end{array}\right.$$
  For the thermal case instead:
  (6) $$Motion_{th}\left(j\right)=\left\{\begin{array}{cc} 1 & \mathrm{if}\;\exists \;\;u\;:\;(s_{1}\left(uT_{s}\right)\geq thr_{2}\;\mathrm{OR}\;\\ & \;\;s_{2}\left(uT_{s}\right)\geq thr_{2}\;\mathrm{OR}\;\\ & \;\;s_{3}\left(uT_{s}\right)\geq thr_{2})\\ 0 & \mathrm{otherwise}. \end{array}\right.$$
  $s_{1}\left(uT_{s}\right)$, $s_{2}\left(uT_{s}\right)$, and $s_{3}\left(uT_{s}\right)$ are the ratios of moving pixels obtained from the three thermal views.
- Ground Truth: The ground truth used to evaluate the performance of our motion detector was obtained based on the manual annotations presented in Section 2.1.3. In particular, the ground truth was built using the sliding window approach. Each window was classified as excluded, as type 1 motion, or as usable. The condition used was the presence of at least a frame in the window which results in being true for one of those categories. The excluded class had the priority, if this was true for at least a frame in the window, the entire window was classified as excluded. If the latter was false then type 1 motion was taken into consideration in the same manner, and lastly if the two above were both false we classified the window as usable.
- Parameters Optimization: the factor $f_{1}$, for the moving pixels detection, and the threshold $thr_{2}$, for the motion classification, were optimized. A leave-one-subject-out cross-validation was used to optimize the two parameters. The approach was chosen considering that environment changes, e.g., environment temperature, blankets type, and position, can influence the parameters values and therefore, the between-baby variability is more important than the within-baby variability. The set of parameters that resulted in the highest balanced accuracy for each fold was considered as a candidate set. The final chosen set was the most selected candidate set. This metric was preferred compared to the classic accuracy due to the imbalance in our two classes (usable was more frequent than type 1 motion). The optimization was performed on the training and testing set, presented in Table 1. This set includes 9 babies and therefore, 9 folds were performed in the cross-validation. Two sets of parameters were empirically chosen for the training and correspond to $f_{1}=\left[4;\;5;\;6;\;7;\;8;\;9;\;10;\;11;\;12\right]$ and $thr_{2}=\left[0.004;\;0.005;\;0.006;\;0.007;\;0.008;\;0.09;\;0.010;\;0.011;\;0.012\right]$. The most chosen set, used in the next steps, was $f_{1}=8$ and $thr_{2}=0.005$, more information on the results can be found in Section 3.

#### 2.2.3. Respiration Rate Estimation

Respiratory signal and rate were both estimated in the windows in which the motion detection step results in the usable category using an adjustment of our previous method [26]. Briefly, first the images of the thermal videos were merged together in a single image plane, resulting in a single video with resolution 180×80, whereas the grayscale videos were processed with the single view available, i.e., videos with resolution 192×256. These two possible frames dimensions will be referred to as M×L. Our method is based on the automatic detection of the pixels containing respiration information. This is performed using the three features presented in [26], improvements were applied to tackle new challenges highlighted by the extension of our dataset and of the acceptable motion.

The changes involve an adaptation of the second feature, Respiration Rate clusters, adapted to overcome the presence of the respiration’s first harmonic and NNS pattern in some of the extended dataset. The third feature (Gradient) was also adapted for the use on visible images, now added to the dataset, and finally the correlation value that indicates which pixels contain the respiration information was increased. More in detail, each pixels’ time domain signal is indicated as $x_{m,l}\left(nT_{s}\right)$, with (m,l) indicating the pixel. Three features were used to find a core-pixel, in each $\hat{j}$th window, which was then employed to find (using a correlation metric) all the helpful pixels that can be combined to compute the respiratory signal, with $\hat{j}=j\;:\;Motion\left(j\right)=0$.

**Figure 3:**
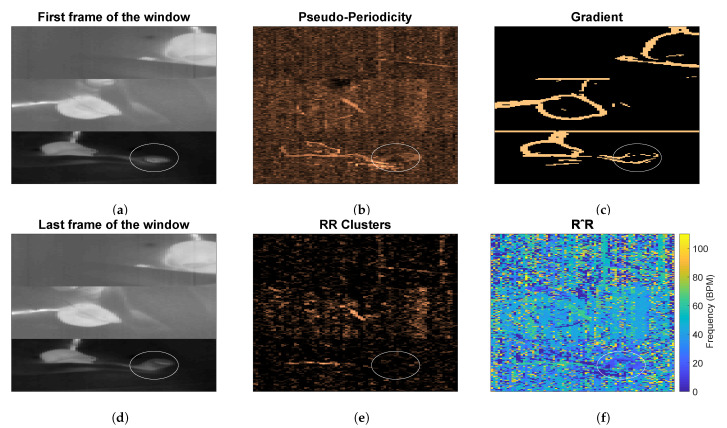
Example of features obtained during a type 2 motion, i.e., arm motion. In (**a**,**d**) the merged thermal images are presented, the circle indicates the position of the baby’s arm where the type 2 motion is happening. The images in (**b**,**c**,**e**) show the three features. While in this case, pseudo-periodicity and gradient are sensitive to the presence of type 2 motion, Respiration Rate (RR) clusters are not, this is due to the $\hat{\mathrm{RR}}$ matrix shown in (**f**) where the arm area can have frequencies equal to zero.

- Pseudo-Periodicity: this first feature is based on the assumption that respiration can be considered a periodic signal. This feature was not changed compared to [26]. A differential filter was used to attenuate low-frequencies resulting in filtered time domain signals called $x_{m,l}^{\prime }\left(nT_{s}\right)$. The signals were zeropadded, reaching a length equal to $N_{z}=120\cdot N$, and multiplied for an Hanning window. Afterwards, a 1D Discrete Fourier Transform (DFT) was used to estimate the spectrum called $y_{m,l}^{\prime }\left(f_{k}\right)$ with $k=0,\;1,\;\ldots ,\;\frac{N_{z}}{2}-1$ and $f_{k}=\frac{k}{N_{z}\cdot T_{s}}\;Hz$. This feature consists of the calculation of the height of the normalized spectrum’s peak. More formally:
  (7) $$q_{m,l}=\frac{\max_{0\leq f_{k}\leq \frac{\left(N_{z}/2-1\right)}{N_{z}\cdot T_{s}}}\underset{}{\left(|y_{m,l}^{\prime }\left(f_{k}\right)|\right)}}{\sqrt{\sum_{f_{k}=0}^{\frac{\left(N_{z}/2-1\right)}{N_{z}\cdot T_{s}}}{|y_{m,l}^{\prime }\left(f_{k}\right)|}^{2}}}.$$
  Each $q_{m,l}$ represent the height of the peak of the spectrum of the pixel in position (m,l), $q_{m,l}$ are elements of the first feature Q.
  This feature is sensitive to the presence of type 2 motion. Regions moving due to this type of motion can generate a big variation in the pixels’ values (depending on the contrast). This variation can, therefore, produce a strong DC component, which will result in a high $q_{m,l}$. The combination with the other features allows us to obtain motion robustness, Figure 3 presents an example during a type 2 motion and the pseudo-periodicity feature is visible in Figure 3b.
- Respiration Rate Clusters (RR Clusters): this feature is based on the observation that respiration pixels are not isolated but grouped in clusters. To automatically identify the pixels of interest more accurately, modifications were introduced to this feature to improve the robustness to the presence of NNS, typical when the infant has the soother, and to cope with the presence of the respiration’s first harmonic. The frequencies corresponding to the local maxima of the spectrum $y_{m,l}^{\prime }\left(f_{k}\right)$ were found and the properties of the harmonic were checked:
  (8) $$h_{m,l}=\underset{lim_{1}<f_{k}<lim_{2}}{arg\;localmax}\left(|y_{m,l}^{\prime }\left(f_{k}\right)|\right),$$
  $h_{m,l}$ is a vector, obtained for the pixel in position (m,l), containing the frequencies of the local maxima in the band of interest, which is identified by $lim_{1}$ and $lim_{2}$ respectively 0.5 and 1.83 Hz. The length of the vector is, therefore, variable and dependent on the spectrum content of each pixel (m,l), this operation was performed using the MATLAB function *findpeaks*. The harmonic properties were checked:
  (9) $$rr_{m,l}=\left\{\begin{array}{cc} h_{m,l}\left(1\right) & \mathrm{if}\;\exists \;\hat{z}>1:|h_{m,l}\left(\hat{z}\right)-2\cdot h_{m,l}\left(1\right)|<\frac{1}{N\cdot T_{s}}\;AND\\ & (y_{m,l}\left(h_{m,l}\left(\hat{z}\right)\right)<y_{m,l}\left(h_{m,l}\left(1\right)\right)\;AND\\ & y_{m,l}^{\prime }\left(h_{m,l}\left(\hat{z}\right)\right)\geq y_{m,l}^{\prime }\left(h_{m,l}\left(1\right)\right))\\ \underset{f_{k}}{arg\;max}\left(|y_{m,l}^{\prime }\left(f_{k}\right)|\right) & \mathrm{otherwise}, \end{array}\right.$$
  $y_{m,l}\left(f_{k}\right)$ is the spectrum of the pixels’ time domain signal calculated as $y_{m,l}^{\prime }\left(f_{k}\right)$ but without applying the differential filter and $h_{m,l}$ is an element of $h_{m,l}$.
  We have, therefore, estimated the main frequency component for each pixel. To avoid erroneous RR estimation caused by higher frequencies components, e.g., caused by NNS, the $rr_{m,l}$ that were higher than $lim_{2}$ were put to zero. Therefore:
  (10) $${\hat{rr}}_{m,l}=\left\{\begin{array}{cc} rr_{m,l} & \mathrm{if}\;rr_{m,l}<lim_{2}\\ 0 & \mathrm{otherwise}. \end{array}\right.$$
  The ${\hat{rr}}_{m,l}$ are elements of $\hat{\mathrm{RR}}$, an example is shown in Figure 3f. The non-linear filter introduced in [26] was applied:
  (11) $$w_{m,l}=\frac{1}{9}\sum_{r=1}^{3}\sum_{o=1}^{3}\left(\frac{1}{exp(\kappa_{1}\cdot |{\hat{rr}}_{m,l}-{\hat{rr}}_{r,o}|/{\hat{rr}}_{m,l})}\right),$$
  where *r* and *o* identify the kernel cell, whereas *m* and *l* indicate the pixel. $\kappa_{1}$ is a constant empirically chosen and equal to 70 as indicated in our previous work [26]. The resulting frame W will map the pixels having similar frequencies around them.
  It should be noted that the ${\hat{rr}}_{m,l}$ on which we imposed the value 0 in Equation (10), will not result in a high $w_{m,l}$, even if there are clusters of zeros in $\hat{\mathrm{RR}}$. This is due to the equation of the filter that with ${\hat{rr}}_{m,l}=0$ will produce NaNs (Not a Number). The same will happen for regions with type 2 motion, where the main frequency component is the DC. This property allowed to avoid type 2 motion regions in the pixel selection phase achieving motion robustness, an example is visible in Figure 3e.
- Gradient: this last feature is based on the assumption that respiration motion can be only visualized at edges. This feature has been modified to make it independent of the setup used:
  (12) $$g_{m,l}=\left\{\begin{array}{cc} 1 & \mathrm{if}\;\sqrt{{\left(\frac{\partial {\bar{a}}_{m,l}}{\partial m}\right)}^{2}+{\left(\frac{\partial {\bar{a}}_{m,l}}{\partial l}\right)}^{2}}>\frac{Range(A)}{\kappa_{2}},\\ 0 & \mathrm{otherwise}, \end{array}\right.$$
  where $\frac{\partial }{\partial m}$ and $\frac{\partial }{\partial l}$ represent the partial derivatives in the two spatial dimensions, $\kappa_{2}$ is an empirical threshold equal to 16, which resulted in identifying the edges of both thermal and grayscale images and A is the series of frames in the $\hat{j}$th window. $\bar{A}$ is an average image representative of the current window $\hat{j}$ evaluated as the average of all the images in A, with elements ${\bar{a}}_{m,l}$. The resulting matrix will be the third feature G. The use of $\bar{A}$ to evaluate the gradient can also ensure robustness to some type 2 motion, whose regions will not be visible in the average image if the motion is transient enough. In the example in Figure 3c the pixels involved in the type 2 motion are still selected in the gradient feature, but RR Clusters ensures the correct pixels are chosen.

The features, Q, W, and G, were then combined together, after being normalized between 0 and 1, by multiplying them and obtaining V. This feature combination was used to identify the core-pixel as:

(13) $$\left(m_{p_{r}},l_{p_{r}}\right)=\underset{\left(m,l\right)}{arg\;max}\left(v_{m,l}\right),$$

where $v_{m,l}$ identifies an element of V. The pixels containing respiration information were then found from this core-pixel based on the Pearson’s correlation coefficient, estimated using a bandpass filtered version of the pixels’ time domain signal. The filter used is a Butterworth bandpass between $lim_{1}$ and $lim_{2}$. In our previous work [26] pixels having a correlation higher than 0.7 with the core-pixel were considered to contain respiration information, this threshold on the correlation value has been increased in the current work considering the reduction in window size and the fact that the accuracy of the correlation estimation depends on the length of the signal. Therefore, the threshold has been set to 0.9 and indicated with $\kappa_{3}$. In particular:

(14) $$p=\left(m,l\right)\;:\;|c_{m,l}|>\kappa_{3},$$

where $c_{m,l}$ is the correlation between the core-pixel $\left(m_{p_{r}},l_{p_{r}}\right)$ and the signal of the pixel in position (m,l), calculated using the MATLAB function *corrcoef*. p will, therefore, be a vector indicating the pixels containing the respiration signal and can have variable dimension depending on the window $\hat{j}$. To calculate the RR and the respiration signal, all the bandpass filtered time domain signals of the pixels in p were combined using an average operation. The RR was calculated from the spectrum of this signal after using a Hanning window, and the RR was estimated as the frequency corresponding to the spectrum’s peak for each window. The same was applied to the CI signal to estimate the reference RR from the waveform. These spectra were then arranged into a Short Time Fourier Transform (STFT).

### 2.3. Evaluation Metrics

Accuracy, balanced accuracy, sensitivity, and specificity were calculated for the test step of the cross-validation and for the validation dataset to obtain unbiased performance results. The RR was compared to the one obtained using the CI. Mean Absolute Error (MAE), Root Mean Square Error (RMSE), and Percentage of correct estimation (PR) [26], considering an accuracy of 3.75 Breaths Per Minute (BPM) caused by the window size, were calculated. We estimated the Percentage of Time used (PT) by calculating the percentage of windows classified as usable by the motion classification step on the number of windows in the included data (which includes also type 1 motion occurrences).

To prove the improved motion robustness of our algorithm, we used the annotations to identify the moments containing only type 2 motion and compared it with the ones containing only stillness. Moreover, the contribution of the NNS segments to the error was also analyzed. The average MAE was obtained in all these windows to analyze their contribution to the final error. In these cases, PT is calculated by considering also the information of the manual annotation on the occurrences of specific events. For example, PT for the segments containing only type 2 motion is calculated considering the number of windows classified as usable by our motion detection and that according to the manual annotation contain only type 2 motion, or PT in the usable segments excluding NNS is evaluated using the number of windows classified as usable and that do not contain NNS according to the manual annotation.

## 3. Results

**Figure 4:**
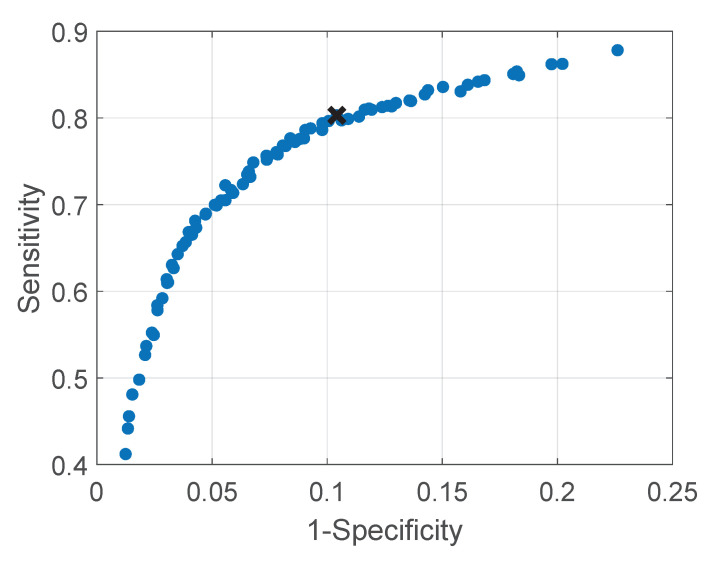
Receiver Operating Characteristics (ROC) curve obtained with the nine folds of the cross-validation by using all the parameters combinations.

The average Receiver Operating Characteristics (ROC) curve for all nine folds obtained from the cross-validation applied on the training and testing set, is presented in Figure 4. The blue points represent the average sensitivity and specificity on all folds for that particular combination of $f_{1}$ and $thr_{2}$, whereas the cross is the average sensitivity and specificity on all folds corresponding to the most chosen parameter set. Table 3 shows the results of accuracy, balanced accuracy, sensitivity, and specificity using the final chosen set of parameters for the testing stage of the cross-validation and for the validation set that was not involved in the training.

The results obtained in the RR detection step are shown in Table 4 and Table 5. The first one shows the MAE obtained in all moments considered usable by our own motion detection step (that includes segments containing NNS) and the error in the moments containing only NNS, whose windows were determined using the manual annotation. Moreover, a comparison between the respiration detection method introduced in our previous work [26], and the modified one introduced in this work is also presented. Table 5 contains the results obtained in all the usable segments excluding the NNS windows on the two sets. Moreover, using the manual annotation, we also show the errors in the windows containing only type 2 motion, and in the ideal moments in which the infants are still.

**Figure 5:**
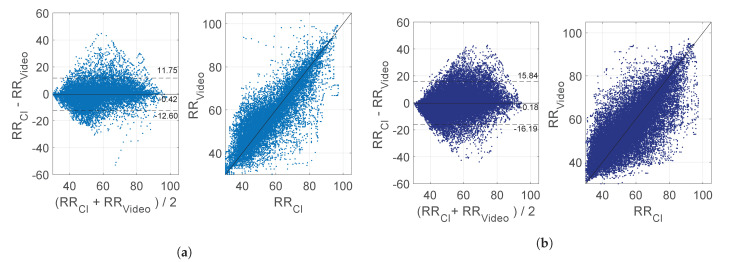
Bland-Altman and correlation plot: (**a**) training and testing set, (**b**) validation set. *RR_CI_* and *RR_Video_* are in BPM.

**Figure 6:**
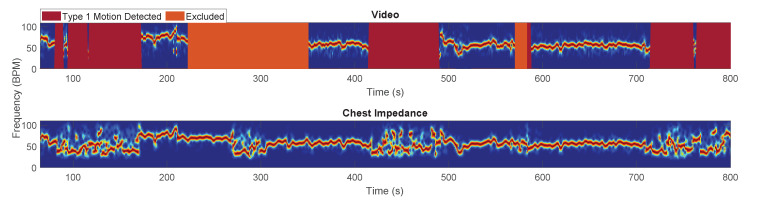
Example of the Short Time Fourier Transform (STFT) obtained using the camera and the Chest Impedance (CI) reference. The noisiness of the reference’s spectrum during type 1 motion shows the sensitivity of the reference to this type of artifact. The excluded segments are due to camera motion.

**Figure 7:**
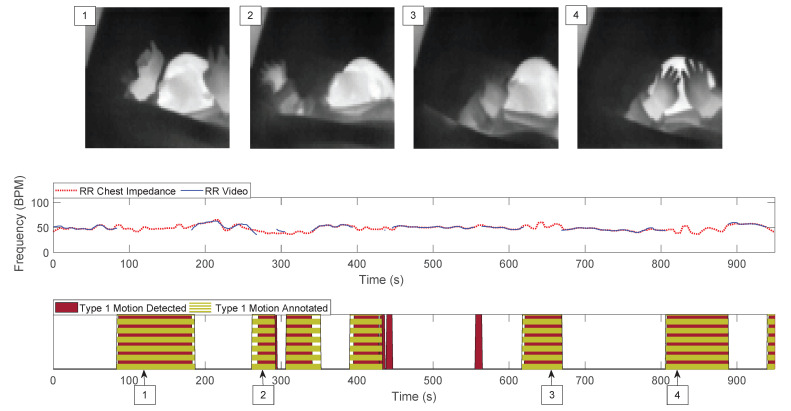
Example of results showing the RR estimated using our cameras and algorithm, and the reference one. The difference in the manual annotation of type 1 motion and the detected one is visible in the bottom plot. Examples of frames during the type 1 motion (infant crying) are also shown.

**Figure 8:**
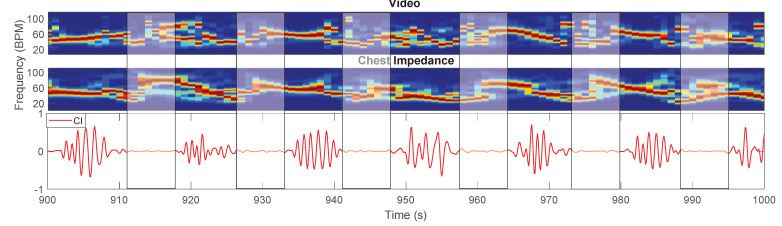
Example of results with periodic breathing. The sudden changes in RR can be seen in the STFTs close to the breathing pauses (indicated using the rectangular boxes with width of 8 s).

Figure 5a,b present Bland–Altman and correlation plots for the training and testing set, and the validation set, respectively, using the RRs in all the usable windows excluding the NNS. The mean bias were −0.42 and −0.18 BPM and the correlation plot shows the agreements between the reference and our estimation with a ρ=0.90 and ρ=0.80 for the training and testing set, and the validation set. Example results are presented in Figure 6, Figure 7 and Figure 8.

## 4. Discussion

Our method for motion robust respiration detection can be used for both thermal and visible modalities, and it does not rely on skin visibility or facial landmark detection. Moreover, it is able to detect motion events that are problematic for respiration monitoring, ensuring a more accurate RR detection and delivering motion information. The manual annotation showed that the RR can be potentially estimated in around 70% of the included data, since the remaining 30% is annotated as type 1 motion. The impossibility to accurately estimate a RR in these segments is a limitation present in all unobtrusive technologies but also in the current monitoring modalities, i.e., CI. An example of the RRs estimated using both camera and CI in the moments annotated and automatically classified as type 1 motion is provided in Figure 6. The sudden noisiness in the spectrum clearly indicates the inaccuracy of the RRs estimation in these segments. Table 5 shows an average PT of around 64% and 70% for the two sets, however, there is considerable variability in the PT between the infants, especially in the training and testing set, as shown by the standard deviation. Infants that are more agitated will have an increased occurrence of type 1 motion, reducing the amount of time usable for RR detection, which can be also lower than 50% (can be partially due to NNS occurrence as well). However, considering that CI is also unable to provide a RR in these cases, the information that the infant is agitated and moving frequently may be much more informative than an inaccurate estimation of RR. In addition, a patient who is moving for a longer period of time is not likely to be in danger due to a serious apnea and, therefore, the motion information itself is giving information about the patient, e.g., the motion could be also linked to the discomfort of the infants [27].

Our motion classification reached an accuracy equal to 88.22% in the training and testing set. It should be noted that the accuracy results are underestimating the real accuracy. The manual annotation was performed by a single author and while the automatic classification is on a second by second basis (due to the sliding window’s slide), the manual annotation tends to overlook particularly short events. An example is visible in Figure 7, the detected label (the result of the automatic classification) can present fast oscillations, whereas the manual annotation is more stable and sometimes stretched compared to the detected label (anticipated starting point and/or postponed ending point). The validation set obtained a lower accuracy result, i.e., 82.52%, this is due to the reduced sensitivity of our motion classification on this dataset. These results could indicate that not enough data were included in our optimization step or that the training dataset is not representative enough. Differences were observed between the two sets in the blanket position, which could end up hiding some of the moving pixels. Whether the infant’s sleeping position plays a role warrants further analysis. Moreover, the motion detection strategy, as it is implemented now, is limited by changes in the distance (between camera and infant) or zoom, however, all infants in our study occupy a similar portion of the image, although small variations are present. The method may need to be optimized for different distances or features in the images could be used to make the method independent of the distance.

Table 4 presents a comparison of the MAE obtained by our previously published method [26] and the adjusted one presented in this work, obtaining an improvement of around 1 BPM on the average MAE. The harmonic problem was particularly noticeable in one of the infants, i.e., infant 8, where the introduction of our adjustment drastically reduced the error (from 7.17 to 1.89 BPM). The NNS is present in less than 5% of the included segments. This is mostly due to the study protocol since hours in which the parents were not in the wards were preferred, as the babies would then spend more time in the bed, but this was not always possible. The percentage of presence of NNS is, therefore, likely underestimated and not completely representative. This percentage could be higher if the parents are in the neonatal ward next to the infant or in home-care because the soother will be given to the baby more often in these cases. The MAE obtained during NNS is reduced in our new implementation, though still higher than the average MAE considering all usable segments. NNS frequencies have been reported to vary and can correspond to the ones of the normal RR or be higher up to 150 sucks per minute [28,29]. Therefore, if the frequencies of NNS are higher than the normal RR range, our algorithm can detect the respiration pixels and correctly estimate the RR. However, if the NNS frequency is inside the respiration band, our method can no longer discriminate between NNS and respiratory signals. This is a limitation present in all methods that automatically identify the region of interest or technologies that monitor the motion in an area, e.g., continuous-wave radars. This problem, particularly important for home-care and babies cared for in open beds, should be further analyzed.

Furthermore, in Table 5, a comparison of the results between the training and testing set, and the validation set, in the usable moments excluding NNS, is provided. The errors are higher in the validation set compared to the training and testing one. We believe this is a consequence of the reduced sensitivity of the motion classification step for the validation set, which leads to the inclusion of segments with type 1 motion in the moments used for the estimation of the RR. Other factors influence the average error, one is the presence of babies breathing with a Periodic Breathing (PB) pattern, a physiological breathing pattern in infants associated with the alternation of normal breathing and breathing pauses [30]. One of the babies in the training and testing set continuously breathes following a PB pattern (infant 3), whereas another baby in the training and testing set (infant 4) and six babies in the validation set (infants 10, 11, 12, 13, 15, and 17) resulted in having segments with a PB pattern. PB pauses have been reported to last 6 to 9 s [31], in our dataset, we observe breathing pauses with a duration of up to 10 s. It becomes evident that by using a window size of 8 s, we will detect a RR in windows that do not contain any respiration-related oscillations. This causes the estimation of the error to be higher than the real one because both our method and the CI will provide an incorrect estimation of the RR, an example is visible in Figure 8. Our method requires the selection of respiration pixels in every window, if there is no respiration information in the video segment, the selected pixels will contain noise. The results are, therefore, also dependent on the length of the breathing pauses which can be different for each baby. This problem needs to be further analyzed considering also apneas, and the number of pixels selected could be used as an indicator to detect the absence of respiration. The PB pattern is, anyway, clearly visible in the time signals, and in the future, methods for cessations of breathing detection such as [32,33] could be used to identify the breathing pauses and remove these from the RR comparison. Moreover, some of the recordings in the validation set (belonging to infants 13 and 14) contain segments in which the respiration motion is not visible due to the blanket position, directly influencing the error. This problem was highlighted also in our previous work [26].

By comparing the errors in the ideal moments where the infants are still and in the moments where type 2 motion occurs, differences can be noted. On average, the MAE during type 2 motion segments is higher than the one during ideal moments, with an increase of 1.16 and 1.97 BPM for training and testing, and validation set, respectively. We believe the cases in which the errors are higher for the type 2 motion may be related to the position of the pixels containing respiration. Our approach is based on the assumption that respiratory pixels are visible on the edge of the blanket and chest/neck area, and type 2 motion, like arm motion or head motion, will not affect our performance. However, this is not always true, like in cases where most of the respiration pixels come from the arm or the head itself, which is happening in some babies’ videos. This is again caused by the blanket covering the main source of respiration signal, i.e., the chest. We can expect this problem to be further reduced in infants in incubators that are not covered. The inclusion of the type 2 motion segments allows to drastically increase the amount of time used for respiration estimation at a cost of a higher error.

The two videos of infants 8 and 9 collected using an RGB camera seem to perform better, yielding lower MAE compared to the other babies (except for infant 1 whose MAE is comparable). However, we believe that conclusions regarding which technology performs best cannot be drawn from this comparison, as such would require a dataset acquired simultaneously with both camera types. Moreover, the RGB videos were not included in the validation set, therefore, the performance of our algorithm on this type of videos should be further analyzed and more data should be included.

Overall, our MAEs and Bland-Altman plots are comparable with studies performed in similar populations, e.g., the work of Villarroel et al. [25] showed a MAE of 4.5 and 3.5 BPM for their training and test set respectively, very similar compared to our 3.31 and 5.36 BPM. Our method, though, can be used on both thermal and RGB/NIR cameras, provides motion information, and does not rely on skin visibility but only on respiration motion being visible. The limits of agreement in Figure 5b are higher than the ones in the training and testing set and higher compared to the results obtained in [25], this is due to a combination of the problems previously described.

Our study provides promising results and highlights possible challenges for neonatal respiration monitoring. In particular, in the cases of babies cared for in an open bed and babies in a home-care environment, the NNS presence and its effect on unobtrusive vital signs solutions should be investigated further, although the presence of the NNS motion itself could indicate the absence of critical situations. Moreover, one of the main limitations of our method, but in general of camera-based solutions, is the respiration motion being hidden by blankets covering the infants. While camera-based solutions provide contextual information undoubtedly usable for the detection of motion, they may also require the fusion with a different technology that would not be affected by this type of problem, such as radar or pressure-sensitive mats, or a clearer protocol for blanket positioning.

## 5. Conclusions

This work presents a combination of a method for motion detection, optimized to detect motion hiding the respiration, and a method for RR detection that, using three features, automatically selects the pixels of interest. The motion robustness achieved thanks to our features, allows us to increment the amount of time used for camera-based respiration detection, including segments that contain limbs or head movements. The test of the cross-validation obtained an accuracy of around 88% in the motion identification. A lower accuracy was obtained in our validation set, indicating that the optimization could be improved. The RR estimation was compared with the chest impedance reference and yielded an average MAE of 3.31 and 5.36 BPM for the training and testing set, and validation set, respectively. The MAE during type 2 motion was higher than the one in the ideal moments of 1.16 and 1.97 BPM for the training and testing set, and validation set, respectively. This proves the motion robustness is improved, but more work is needed to achieve continuous unobtrusive respiration monitoring. Therefore, limitations on the use of camera-based solutions in a neonatal ward environment are highlighted in this study, i.e., the PB influence of the errors, the blanket covering respiration motion, and the NNS presence. This method can be used for different camera modalities and does not require skin visibility.

## Figures and Tables

**Table 1:** Infants’ data for the training and testing set (indicated with T&T) and the validation set (indicated with V).

Infant	Video Type	Gestational Age (weeks + days)	Postnatal Age (days)	Sleeping Position	Duration (hours)	Set
1	Thermal	26w 4d	59	Supine	2.98	T&T
2	Thermal	38w 5d	3	Supine	2.74	T&T
3	Thermal	34w 1d	16	Supine	2.93	T&T
4	Thermal	26w 3d	59	Prone	3.16	T&T
5	Thermal	39w	2	Lateral	3.05	T&T
6	Thermal	40w 1d	6	Supine	2.95	T&T
7	Thermal	40w 2d	1	Lateral	0.92	T&T
8	RGB	36w	47	Supine	0.30	T&T
9	RGB	30w	34	Supine and Lateral	0.57	T&T
10	Thermal	26w 4d	77	Supine	2.94	V
11	Thermal	26w 4d	77	Supine	2.97	V
12	Thermal	33w 4d	5	Supine	2.97	V
13	Thermal	34w 2d	9	Supine	2.87	V
14	Thermal	32w 2d	11	Supine	2.96	V
15	Thermal	35w 1d	8	Supine	2.94	V
16	Thermal	38w 1d	2	Supine	3.00	V
17	Thermal	27w 5d	16	Supine	2.96	V

**Table 2:** Labels used for the manual annotation.

	Annotation Labels	Subcategories and Details
**Included**	**(i) Infant activity**	Still Type 1 motion (motion including chest/torso area) Type 2 motion (motion involving limbs or head)
	**(ii) NNS**	-
**Excluded**	**(iii) Interventions**	includes both parents and caregivers interventions
	**(iv) Other**	Someone in the background Baby out of bed Camera motion Unsuitable view

**Table 3:** Average performance of the motion detection step for all the babies of the training and testing, and the validation set using the chosen parameters.

	Accuracy	Balanced Accuracy	Sensitivity	Specificity
**Training and testing set**	88.22%	84.94%	80.30%	89.58%
**Validation Set**	82.52%	77.89%	66.85%	88.93%

**Table 4:** Average and standard deviation of Mean Absolute Error (MAE) and percentage of used time (PT) on all babies of the training and testing set for the previous version of method [26] and the current one presented in this work, in the windows classified by the motion detector as usable. We further show the contribution of the Non-Nutritive Sucking (NNS) to the overall error (these segments were obtained using the manual annotation).

	Previous Version of Method [26]		Current Version of the Method	
	Usable	NNS Only	Usable	NNS Only
**MAE (BPM)**	4.54 ± 1.82	9.39 ± 3.68	3.55 ± 1.63	7.11 ± 4.15
**PT**	68.59% ± 13.29%	4.59% ± 6.93%	68.59% ± 13.29%	4.59% ± 6.93%

**Table 5:** Results of the two sets in the segments classified as usable by our motion detector excluding the NNS windows, obtained thanks to the manual annotation. The errors in the windows containing type 2 motion and moments where the infants were still are also included. MAE and RMSE are in Breaths Per Minute (BPM).

	Infant	Usable Excluding NNS				Type 2 motion Only		Still Only	
		MAE	RMSE	PR	PT	MAE	PT	MAE	PT
**Training and testing**	**1**	1.86	3.34	83.61%	70.38%	1.57	27.92%	1.51	34.61%
	**2**	2.87	3.97	73.71%	40.60%	2.56	20.90%	2.64	13.02%
	**3**	6.30	8.09	39.44%	67.83%	6.32	39.23%	6.28	24.38%
	**4**	4.43	6.21	60.16%	72.75%	4.99	44.09%	2.49	20.39%
	**5**	5.04	7.61	56.44%	40.22%	4.84	29.24%	2.24	5.35%
	**6**	2.97	4.73	71.34%	66.74%	3.70	29.96%	1.94	31.69%
	**7**	2.80	4.15	72.08%	46.16%	2.57	30.28%	0.70	4.61%
	**8**	1.89	3.40	88.63%	89.71%	1.76	11.47%	1.91	77.84%
	**9**	1.62	2.70	85.55%	81.60%	2.88	24.16%	1.08	56.76%
	**Average**	**3.31**	**4.91**	**70.11%**	**64.00%**	**3.47**	**28.58%**	**2.31**	**29.85%**
	**± sd**	**± 1.61**	**± 1.94**	**± 15.84%**	**± 17.82%**	**± 1.62**	**± 9.56%**	**± 1.62**	**± 24.22%**
**Validation**	**10**	4.46	6.62	61.41%	63.62%	5.52	34.40%	2.44	22.78%
	**11**	3.79	5.54	64.96%	55.55%	4.01	34.62%	2.27	12.29%
	**12**	6.23	7.98	38.98%	68.20%	5.98	33.70%	6.60	23.35%
	**13**	6.29	8.51	44.00%	69.53%	6.30	51.04%	3.59	6.13%
	**14**	6.89	9.56	47.37%	73.38%	7.35	44.73%	4.58	18.00%
	**15**	4.75	6.65	54.11%	78.86%	4.83	42.08%	4.39	26.81%
	**16**	4.09	5.73	60.97%	76.84%	4.39	28.92%	3.21	30.73%
	**17**	6.40	8.78	47.79%	71.22%	7.64	40.14%	3.15	19.60%
	**Average**	**5.36**	**7.42**	**52.45%**	**69.65 %**	**5.75**	**38.71%**	**3.78**	**19.96%**
	**± sd**	**± 1.21**	**± 1.49**	**± 9.35%**	**± 7.47%**	**± 1.32**	**± 7.14%**	**± 1.40**	**± 7.90%**

## Data Availability

The data are not publicly available due to privacy reasons.
